# Using HPV-meta for human papillomavirus RNA quality detection

**DOI:** 10.1038/s41598-022-17318-5

**Published:** 2022-07-29

**Authors:** Agustin Ure, Dhananjay Mukhedkar, Laila Sara Arroyo Mühr

**Affiliations:** 1 Department of Laboratory Medicine, Karolinska Institutet, 141 86, Stockholm, Sweden; 2 Hopsworks AB, Medborgarplatsen 25, 118 72 Stockholm, Sweden

**Keywords:** Computational biology and bioinformatics, Tumour virus infections

## Abstract

In the era of cervical cancer elimination, accurate and validated pipelines to detect human papillomavirus are essential to elucidate and understand HPV association with human cancers. We aimed to provide an open-source pipeline, “HPV-meta”, to detect HPV transcripts in RNA sequencing data, including several steps to warn operators for possible viral contamination. The “HPV-meta” pipeline automatically performs several steps, starting with quality trimming, human genome filtering, HPV detection (blastx), cut-off settlement (10 reads and 690 bp coverage to make an HPV call) and finishing with fasta sequence generation for HPV positive samples. Fasta sequences can then be aligned to assess sequence diversity among HPV positive samples. All RNA sequencing files (n = 10,908) present in the cancer genome atlas (TCGA) were analyzed. “HPV-meta” identified 25 different HPV types being present in 488/10,904 specimens. Validation of results showed 99.98% agreement (10,902/10,904). Multiple alignment from fasta files warned about high sequence identity between several HPV 18 and 38 positive samples, whose contamination had previously been reported. The “HPV-meta” pipeline is a robust and validated pipeline that detects HPV in RNA sequencing data. Obtaining the fasta files enables contamination investigation, a non very rare occurrence in next generation sequencing.

## Introduction

Human papillomaviruses (HPVs) are a group of double-stranded DNA viruses which comprise up to 222 different types, with new types being continuously identified^1–4^. About 12 HPV types are nowadays classified as oncogenic, high-risk HPV genotypes, and persistent infection of these oncogenic HPV types is known to be a necessary cause for cervical cancer^5^. Furthermore, other cancers as oropharyngeal, anal, penile, vaginal and vulvar carcinomas, have also been associated and are known to be caused by high-risk HPV types in the majority of their cases^6^.

Traditionally, tests for HPV detection are based on broad-spectrum signal amplification assays followed by rapid high-throughput target-amplification assays. Nowadays, next generation sequencing (NGS) has enabled scientists to move beyond simply detecting presence/absence of HPVs and provide a more detailed insight of the HPV sequence and infection.

Metagenomics studies of viral DNA have systematically found HPVs in the described-above tumors, even in cases where HPV had been reported as “apparently negative” when tested with non-NGS methods^7–11^. Another cancer type, non-melanoma skin carcinoma, has also revealed a plethora of human papillomavirus types when skin tumors have been DNA sequenced^2,3,10,12^. However, detection of HPV DNA does not necessarily mean that an HPV infection has been detected^13^ and contrary to the other cancer types, whole transcriptome studies have rarely detected HPV in skin tumors^14,15^. Investigation of viral transcription is important, as transcription of viral genes is necessary for viral pathogenicity, as demonstrated in head and neck cancers^16,17^. Considering the global aim set by the WHO towards elimination of cervical cancer, it seems essential to understand the biology of human papillomavirus by performing metatranscriptomic analysis.

Although NGS has been extensively used in HPV research, there is a higher risk of possible cross-contamination among specimens when compared to non-sequencing methods (due to the high throughput achieved when sequencing), as well as a lack of standardization and quality guidelines when considering sequencing analysis^18^. The International HPV Reference Center already reported existence of 114 possible chimeras and 13 taxonomy/naming errors when performing a GenBank screening analysis of HPV deposited sequences in 2021^19^ and several authors have reported presence of HPV contamination among different studies (e.g. HPV 38 in endometrial samples, HeLa nucleic acid contamination leading to misidentification of HPV 18)^20,21^. Well-validated HPV pipelines are needed to prevent database contamination and false calling to continue with quality and order in HPV research.

The present study aimed to develop a robust open-source HPV pipeline, “HPV-meta” (https://github.com/hpvcenter/HPV-meta) to detect HPV transcripts in RNA sequencing data which could further provide information to operators and warn for possible HPV contamination. To achieve this aim, a well-known and validated public database, the cancer genome atlas (TCGA, https://www.cancer.gov/tcga) was used for pipeline development and validation purposes. TCGA is a collaboration between the National Cancer Institute and the National Human Genome Research Institute and contains molecularly characterization of over 20,000 primary cancers and matched normal specimens spanning 33 cancer types (https://www.cancer.gov/tcga, accessed on 2021-08-10).

## Methods

For this study, we downloaded all RNA sequencing (RNA-seq) datasets from the TCGA portal belonging to primary tumors (n = 9774), metastases (n = 394), recurrent tumors (n = 47) and solid normal tissues (n = 693) to develop and validate “HPV-meta”, a pipeline aiming to detect HPV transcript in RNA sequencing data. All research was performed in accordance with relevant guidelines/regulations.

By using the GDC transfer tool, we downloaded 10,908 bam files comprising around 90 terabyte of sequencing data. For each of the bam files, we downloaded the corresponding metadata, including the specimens and clinical data (sample type -primary tumor, control, metastasis-, ICD-10, tissue or organ of origin and primary diagnosis).

**Figure 1:**
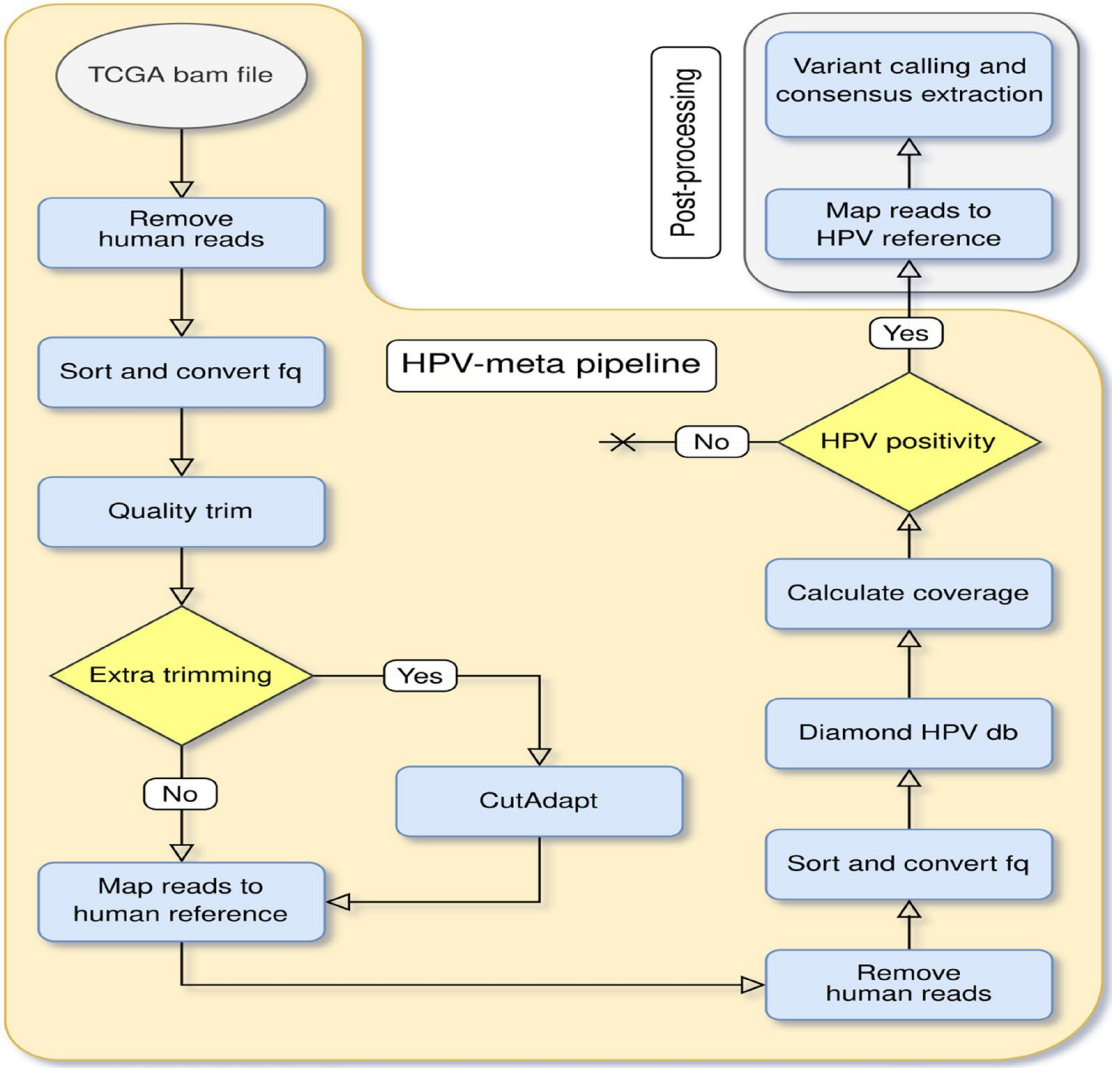
“HPV-meta” pipeline for detecting HPV transcripts in RNA sequencing data. Flowchart describing the pipeline steps included in “HPV-meta”. The pipeline includes removal of human reads, sort and conversion to fq files (samtools v. 1.10), quality trimming (Trimmomatic v. 0.39 (https://github.com/usadellab/Trimmomatic)^22^ extra trimming (needed for specific library preparation kits, e.g: removing 3 bp of R2 from libraries prepared with the Smarter stranded total RNA-seq kit from Takara, USA) performed with Cutadapt v. 3.3)^23^ re-mapping to human reference genome (double human cleaning using Nextgenmap v. 0.5.5)^24^ filtering out of human reads, mapping non-human reads to an HPV protein database (Diamond v. 2.0.7)^25^ coverage calculation and, if HPV positivity is present, a fasta file is generated by mapping the reads to HPV genome references and subjecting them to variant calling using GATK v. 4.2.3.0^26^ (Image created using https://app.diagrams.net/ and Inkscape v. 1.1, https://inkscape.org/).

In order to process the large amount of sequencing data, we developed an automated pipeline called “HPV-meta” for HPV detection in RNA sequencing specimens. “HPV-meta'' performs several steps including quality trimming, human genome filtering, HPV detection, cut-off settlement and fasta sequence generation (Fig. 1). The fasta sequence is intended for users to inspect possible contamination. Be aware that the whole complete sequence may not be obtained, as it is RNA data (non-coding regions will not appear if DNA is not present).

The pipeline and all the tools used for the data processing and analysis are openly accessible in the github repository (https://github.com/hpvcenter/HPV-meta). For this study, the code was integrated onto the Spark and Hadoop environment but it can definitely be used in other environments as well.

A detailed description of the different steps performed by “HPV-meta” is provided as follows.

### Filtering human genome sequences

This step is recommended when starting with sequencing reads that were already aligned to a human reference genome. If the user starts with raw data (fastq files), this step must be omitted, and files should be subjected to quality filtering directly.

A close look at the original bam files included in the TCGA database (n = 10,908) revealed that all bam files were mapped to databases containing a human reference genome as well as other viral sequences. HPV was included in some of those databases. Therefore, the “HPV-meta” pipeline starts by using pysam v. 0.16.1 (htslib/bcftools v. 1.10.2 and samtools v. 1.10) with a bed file containing human chromosomes and genomic regions to filter out the mapped human reads but not the viral sequences. Non-human reads remain in the newly created bam file (file_name_NH.bam), which is then sorted and converted to a fastq file (file_name_NH.fq.gz) using pysam.

### Quality filtering and human re-filtering

To obtain high quality non-human reads, “HPV-meta” uses Trimmomatic v. 0.39 (https://github.com/usadellab/Trimmomatic)^22^. Parameters for Trimmomatic are as follows: -SE -phred 33 -leading 3 -trailing 3- slidingWindow:4:15 and-minlen:18.

In the case of the TCGA, none of the bam files belonged to samples subjected to library preparation kits that needed extra quality trimming steps. However, as library kits are expanding, the “HPV-meta” pipeline includes the step of “extra trimming”, using cutadapt version 3.3. As an example, when performing library preparations using the SMARTer® Stranded Total RNA-Seq Kit v2-Pico Input Mammalian library preparation guide (Takara, US), it is required to trim/remove the first 3 nucleotides from every R2 read for the RNA sequencing fastq-files^23^. The operator can choose to use or to omit this step.

High quality reads are afterwards re-screened against the human reference genome version GRCh38 using Nextgenmap v. 0.5.5^24^. The program is run under default settings, except for -i 0.95, -R 0.75 and–silent-clip. Reads that map to the human reference genome (if they align with more than 95% identity over 75% of their length to the human genome) are filtered out, and the corresponding bam files with no human sequences are then sorted and converted to fastq files again (File_ID_non-human_quality.fq). We highly recommend filtering human sequences twice when users start with bam/sam files coming from public databases as the human reference genomes used previously may not be the latest update.

### HPV detection and cut-offs

High quality non-human reads are queried against all HPV protein sequences included in the PaVE database (Papillomavirus Episteme, https://pave.niaid.nih.gov/, accessed on 2021-08-17), including all protein sequences from HPV reference and non-reference genomes), using the open source software Diamond v. 2.0.7^25^ using blastx with default parameters and –top 1.

Positivity of HPV is only considered if at least 10 reads are present for one HPV type and 690 bp of its genome are covered. The cut-off is based on previous validated cut-offs used for HPV detection on total nucleic acid extracted specimens (cut-off 750 bp,  ~ 10% of total genome)^7,8,18^. As the HPV genome comprises around 8000 bp and approximately 1000 of them are non-coding, the “HPV-meta” pipeline has reduced the number of bp to be covered (from 750 to 690 bp), in order to maintain a similar % of minimum coverage for positivity calling. The “HPV-meta” pipeline calculates the coverage for each sequencing file and reports the total coverage as well as the coverage for each HPV gene. HPV detection will only be reported for one HPV type per specimen. In the case of multiple HPV infection within a sample, the HPV type with the highest number of unique reads will be reported. In the case that 2 HPV types present the same number of unique reads, the type which presents a higher coverage is selected.

### HPV fasta files

The “HPV-meta” pipeline generates a consensus fasta file for each HPV type classified as positive within a specimen. High quality non-human reads fastq files (File_ID_non-human_quality.fq) are queried against a database of known HPV sequences including all HPV genomes officially established by the International HPV Reference Center (n = 222 officially established HPV types, https://www.hpvcenter.se, accessed on 2021-08-17), together with complete genome sequences from HPV types that are not officially established yet (n = 222, https://pave.niaid.nih.gov, accessed on 2021-08-17), using NextGenMap^24^ under the same settings as described above. For each specimen, reads mapping to the HPV genotype previously detected with diamond are filtered for further analysis.

Resulting BAM files are then processed through a quality control and left aligned using the The Genome Analysis Toolkit (GATK) v. 4.2.3.0, LeftAlignIndels module. The HPV genomes are then genotyped by GATK HaplotypeCaller^26^ SNP and indel calls are made and hard filtered, following GATK Best Practices. All variant calls met the following conditions: QualbyDepth < 2.0, FisherStrand > 60.0, Root Mean Square Mapping Quality < 40.0, Mapping Quality Rank Sum Test <  −  12.5 and Read Pos Rank Sum Test <  −  8.0 to avoid strand biases, inflation when there was deep coverage, false calls at the end of the reads and low-quality variant calls. Since the GATK Haplotype Caller uses the HPV reference sequences as a template and annotates mutations/indels on them, the actual coverage (at least five reads) for each consensus was saved as a bed file using covtobed v. 1.3.1 (https://github.com/telatin/covtobed). The bed file was used later to generate a fasta file using bedtools v. 2.30.0, in which the regions of the genome not covered (< 5 reads) were marked with “N”s (instead of having the reference nucleotides in not covered positions).

### Analysis of sequence identity

It is well reported in literature that for each HPV type, extensive sequence diversity is observed when analyzing HPV positive samples. Around 84.5% of women infected with HPV isolates do carry unique HPV sequences within their cervical samples^27,28^. With the generated fasta sequences, operators can for example compare the sequence identities obtained for one HPV type in one sample, with the rest of sequences presenting the same HPV type but detected in other specimens. The present study includes an analysis of sequence identity for all HPVs that were present in at least 30 samples (HPV 16, 18 and 38).

We would like to note that 100% identity is not a final proof of contamination, as different women may be infected with the same HPV type and identical sequence. However, isolates showing 100% nucleotide identity should be considered as “flagged” for the operator in order to have a closer look if needed.

## Results

A total of 10,908 bam files were downloaded from the TCGA database, and subjected to HPV detection using the “HPV-meta” pipeline. Four sequencing files (all corresponding to primary tumors) could not be processed due to failure/corruption in the original bam files and therefore the analysis was made in 10,904 sequencing files (9770 primary tumors, 394 metastases, 47 recurrent tumors and 693 solid normal tissues).

All bam files belonged to specimens located at 137 different organs, with breast (n = 1203), kidney (n = 1019), lung -upper lobe- (n = 642), thyroid gland (n = 564) and endometrium (n = 561) being the organs presenting the most number of specimens.

### HPV detection

The “HPV-meta” pipeline identified 488 HPV positive specimens among the 10,904 bam files (4.5%) according to pipeline cut-offs. A total of 25 different HPV types were detected among the studies included within the TCGA, with HPV 16 (261/488), HPV18 (72/488) and 38 (41/488) being the types most commonly found (Table 1).

**Table.1:** HPV types detected in RNA sequencing data from TCGA using “HPV-meta”.

HPV type	Tissue or organ of origin	Total positive samples
HPV16	Cervix uteri (171); Tonsil, NOS (30); Base of tongue, NOS (7); Tongue, NOS (6); Cerebrum (4);	261
	Overlapping lesion of lip, oral cavity and pharynx (4); Bladder, NOS (4); Larynx, NOS (3);	
	Liver (3); Oropharynx, NOS (2); Endometrium (2); Breast, NOS (2); Prostate gland (2);	
	Upper lobe, lung (2); Connective, subcutaneous and other soft tissues of lower limb and hip (2);	
	Hypopharynx, NOS (2); Hard palate (2); Lower lobe, lung (1); Lower gum (1); Mouth, NOS (1);	
	Floor of mouth, NOS (1); Kidney, NOS (1); Pleura, NOS (1); Posterior wall of oropharynx (1);	
	Sigmoid colon (1); Skin, NOS (1); Head of pancreas (1); Brain, NOS (1); Upper Gum (1);	
	Gum, NOS (1)	
HPV18	Cervix uteri (40); Kidney, NOS (6); Sigmoid colon (6); Bladder, NOS (4); Ovary (3);	72
	Cardia, NOS (2); Cecum (2); Colon, NOS (2); Ascending colon (1); Body of stomach (1);	
	Descending colon (1); Gastric antrum (1); Liver (1); Rectum, NOS (1); Transverse colon (1)	
HPV38	Endometrium (41)	41
HPV45	Cervix uteri (23); Endometrium (2); Liver (2); Lateral wall of bladder (1); Thyroid gland (1)	29
HPV33	Cervix uteri (9); Tonsil, NOS (3); Overlapping lesion of lip, oral cavity and pharynx (2);	18
	Tongue, NOS (2); Base of tongue, NOS (1); Floor of mouth, NOS (1)	
HPV35	Cervix uteri (6); Liver (3); Base of tongue, NOS (2); Kidney, NOS (1); Tonsil, NOS (1)	13
HPV58	Cervix uteri (8); Cerebrum (1)	9
HPV52	Cervix uteri (8); Posterior wall of bladder (1)	9
HPV31	Cervix uteri (7)	7
HPV39	Cervix uteri (5)	5
HPV51	Cervix uteri (1); Kidney, NOS (1); Retroperitoneum (1)	3
HPV59	Cervix uteri (3)	3
HPV30	Cervix uteri (1); Upper lobe, lung (1)	2
HPV68	Cervix uteri (2)	2
HPV70	Cervix uteri (2)	2
HPV56	Cervix uteri (1); Lateral wall of bladder (1)	2
HPV73	Cervix uteri (2)	2
HPV2	Breast, NOS (1)	1
HPV69	Cervix uteri (1)	1
HPV-mSK041	Ovary (1)	1
HPV133	Corpus uteri (1)	1
HPV26	Cervix uteri (1)	1
HPV155	Brain, NOS (1)	1
HPV94	Kidney, NOS (1)	1
HPV6	Anterior wall of bladder (1)	1

HPV types detected in TCGA RNA sequencing files. Number in brackets corresponds to number of specimens for each organ/tissue of origin.

### HPV detection validation

Results from HPV type detection obtained with the “HPV-meta” pipeline were compared to the HPV mapping hits present in the original bam files from the whole TCGA collection for validation purposes (as the database used in the alignment already contained sequences from most HPV types).

All HPV types detected by “HPV-meta” were confirmed within the specimens, except for 2 samples where the genotypes detected with both approaches did not agree. One specimen (cervical primary tumor) showed HPV 52 in the original bam file while the “HPV-meta” revealed HPV 58. Further analysis on original bam files revealed presence of both genotypes, with HPV 52 showing 23 reads and HPV 58 19 reads. The “HPV-meta” pipeline revealed presence of HPV 52 (16 unique reads) and HPV 58 (16 unique reads) and HPV 58 was selected as the dominant HPV type due to higher coverage (690 nt). The other specimen (ovarian primary tumor) showed HPV-mCG3 in the original bam file (15 reads) while the “HPV-meta” revealed presence of HPV-mSK041 (21 reads, coverage 1119 nt). All specimens classified as negative by the “HPV-meta” pipeline have also been reported negative in the TCGA studies.

### HPV coverage

The “HPV-meta” pipeline provided HPV coverage for all HPV positive specimens, revealing that HPV16 showed the highest median coverage when detected in gum, upper gum, oropharynx, base of tongue and tonsil specimens (6870, 6840, 6625, 6231 and 6148 nt covered, respectively, which corresponds to 86.90—77.76% of total genome coverage) followed by HPV 6 detected in anterior wall of bladder (6162 nt covered, 77.06% of total genome coverage), when compared to other HPV types and locations (Supplementary Table S1).

Sequencing reads from a total of 22 organs/tissues of origin where HPV positivity was detected, showed less than 2000 nt coverage (about < 25% total genome coverage) for all HPV types detected at these locations: brain and cerebrum, liver, colon (including ascending, transverse, descending, sigmoid and unspecified), corpus uteri, mouth and floor of mouth, posterior wall of oropharynx, cecum, cardia, thyroid gland, skin, pleura and lower lobe of lung, head of pancreas, prostate gland, ovary and connective, subcutaneous and other soft tissues of lower limb and hip (Supplementary Table S1).

The Supplementary Table S1 (available online) provides the number of total HPV reads and the HPV median coverage (both total and gene coverage), for each HPV type detected in a different tissue/organ location from all RNA sequencing data from TCGA.

### Analysis of sequence identities

**Figure 2:**
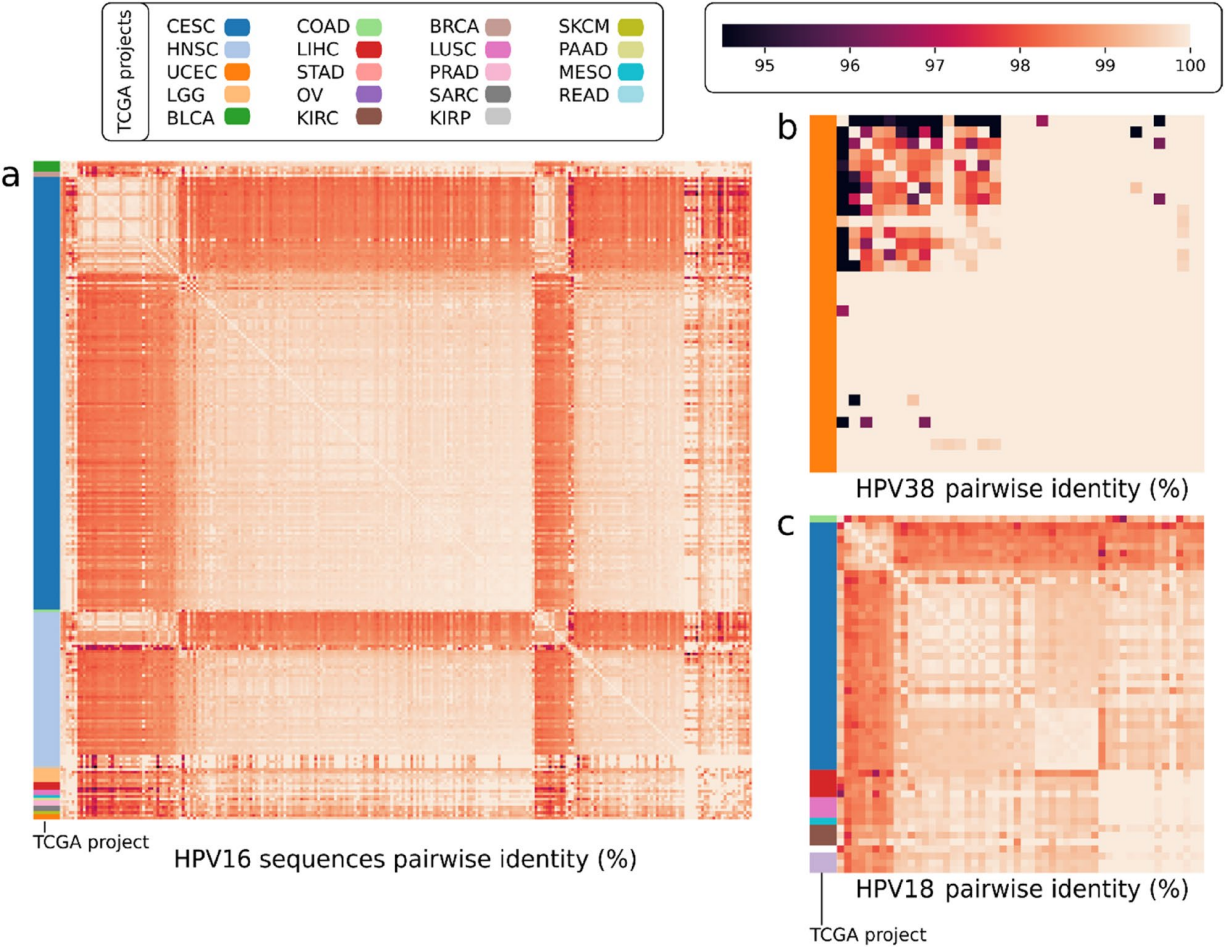
HPV pairwise comparison among HPV positive specimens detected in TCGA. Pairwise comparison performed for HPV16 positive RNA sequences in the TCGA database (**a**), for HPV 38 sequences (**b**) and for HPV 18 sequences (**c**). BLCA: Bladder Urothelial Carcinoma; BRCA: Breast Invasive Carcinoma; CESC: Cervical Squamous Cell Carcinoma and Endocervical Adenocarcinoma; COAD:Colon Adenocarcinoma; HNSC: Head and Neck Squamous Cell Carcinoma; KIRC: Kidney Renal Clear Cell Carcinoma; KIRP: Kidney Renal Papillary Cell Carcinoma; LGG: Brain Lower Grade Glioma; LIHC: Liver Hepatocellular Carcinoma; LUSC: Lung Squamous Cell Carcinoma; MESO: Mesothelioma; OV: Ovarian Serous Cystadenocarcinoma; PAAD: Pancreatic Adenocarcinoma; PRAD: Prostate Adenocarcinoma; READ: Rectum Adenocarcinoma; SARC: Sarcoma; SKCM: Skin Cutaneous Melanoma; STAD: Stomach Adenocarcinoma; UCEC: Uterine Corpus Endometrial Carcinoma. (Image created using Python v. 3.8.10 and Seaborn library v. 0.11.2, https://seaborn.pydata.org/).

Analysis of sequence identity was performed for HPVs that were present in at least 30 samples (HPV 16, 18 and 38). Within the HPV 16 sequences, a total of 245/274 sequences were aligned (29/274 sequences were excluded due to fasta generation failures) and 30,012 pairwise comparisons were done (245 × 245/2). Up to 2078.5/30,012.5 comparisons showed 100% identity, translating into 6.93% sequences showing 100% identity to other HPV 16 sequences.The minimal identity detected among sequences was 94.59% (Fig. 2a).

A total of 32 HPV 38 sequences were analyzed. Minimum identity detected among the HPV 38 sequences was 89.23%, and 100% identity was detected in 83.2% of total counts (852/1024). Clear clusters with 100% identity were detected for this HPV type (Fig. 2b). For HPV 18, 52 sequences were analyzed, comprising 2704 pairwise comparisons (52 × 52), with 13.17% (356/2704) showing 100% identity. Minimum identity was detected at 96.47% (Fig. 2c).

## Discussion

The present study described “HPV-meta”, the first open-source pipeline aiming to specifically detect HPV transcripts in RNA sequencing data and provide further HPV type sequences to inspect for possible viral contamination among samples. We report the description and validation of the pipeline using the TCGA database including almost 11,000 RNA sequencing files.

Strengths of the study include: (a) Using TCGA database, a well validated (as well as exploited) database, that provided no less than 10,904 RNA sequencing files from 137 different organs, spanning 33 cancer types. Open science, data sharing and collaboration is essential for understanding any research question; (b) screening against an updated and complete HPV database including all officially established HPV types as well as the non-official types; and (c) the possibility of generating fasta files (obtain the HPV sequence) that can be used to compare sequence identity among samples presenting the same HPV type within a study. This is due to the singularity of the HPV genome, mostly covered by transcribing ORFs (> 85% of total HPV genome is covered by coding genes).

Weakness of the study may include the single HPV result obtained from the pipeline. The pipeline was designed to report only one HPV type in case of multiple HPV infection. However, the information about the different HPV types detected, the number of unique reads and the coverage is saved for every positive HPV. The operator may choose to change the code in order to report all HPV types detected. Furthermore, “HPV-meta” targets only HPV (no other viruses and/or microorganisms), however, changing the database and including the desired microorganisms´ sequences may enable the pipeline to potentially detect other microorganisms.

“HPV-meta” identified HPV in 488/10,904 specimens and HPV was present in 46/137 organs. For some cancer forms, like cervical cancers, it is already known that they will regularly contain HPV transcripts from the E6 and E7 viral oncogenes and these cancer forms did serve as a positive control in this project. Organs with HPV-associated cancers showed the highest number of specimens positive for HPV as expected (cervix uteri (n = 291/309), endometrium (n = 45/561) and tonsils (n = 34/41)), but HPV was also detected in non-HPV associated tumors (e.g. 7/683 cerebrum/brain tumors). To validate the results, we examined all original bam files from the TCGA as they had the HPV mapping information and compared those results with the results from “HPV-meta”. There was almost a 100% agreement (99.98%) with only 2/488 HPV positive samples not reporting the same genotype. The disagreement of one of them (cervical tumor) could be explained due to a multiple infection of 2 genotypes presenting the same number of reads in the same sample. The second disagreement occurred in one ovarian tumor where 2 different non-officially HPVs were reported. While the type detected in the original bam files corresponded to HPV-mCG3 (non-official HPV sequence submitted in 2012), the “HPV-meta” revealed presence of HPV-mSK041 (non-official HPV sequence submitted in 2018, and therefore not present in the original database). It can be thus confirmed that “HPV-meta” could detect all HPVs with no obstacle.

Possible sequencing contamination is a weakness from NGS and operators must be highly experienced when performing sequencing protocols. Possible contamination within samples has already been reported for TCGA specimens (HPV 38 in endometrial samples and HeLa nucleic acid contamination leading to misidentification of HPV 18 in non-cervical cancers)^20,21^. In agreement with these authors, HPV 38 was detected in 41 samples, and all of them belonged to the same study (endometrium), which should cause at least suspicion about possible contamination. Fasta generated alignments and pairwise comparisons showed total sequence identity among most of the sequences (> 80%) for HPV type 38, raising a flag for possible contamination. The same scenario occurred in some TCGA studies for HPV 18. Use of positive controls may be necesary when performing NGS sequencing, but negative controls are a must.

In conclusion, the “HPV-meta” pipeline has shown to be an open-source pipeline which accurately detects HPV in RNA sequencing data with validated cut-offs. Obtaining the fasta files adds the possibility of contamination investigation, a non-very rare occurrence in next generation sequencing which should always be considered.

## Supplementary Information

Supplementary Information.

## Data Availability

All files used for this study are available at the public database the cancer genome atlas (TCGA). “HPV-meta” is publicly available at https://github.com/hpvcenter/HPV-meta.

## Abbreviations

HPV: Human papillomavirus
PAVE: Papillomavirus Episteme
TCGA: The Cancer Genome Atlas
